# Covid-19 Associated Psychosis in Children and Young Person – a Systematic Review of Case Reports

**DOI:** 10.1192/bjo.2025.10193

**Published:** 2025-06-20

**Authors:** Amey Angane, Victoria Fernandez Garcia De Las Heras

**Affiliations:** South West London St George’s Mental Health NHS Trust, London, United Kingdom

## Abstract

**Aims:** The primary objective of this descriptive systematic review of case reports is to describe the clinical commodities, presentations and outcomes in children and adolescents presenting with onset of non-delirious psychosis during or shortly after a SARS-CoV-2 infection and to find out statistically various other factors that might be linked to demographics of young people. The review also explores if the clinical presentation of the Covid-19 psychosis is different from early onset non-organic psychosis occurring in children and adolescents.

**Methods:** On 23 September 2023, the author searched six electronic databases including PubMed, Scopus, Web of Science, PsycInfo, Google Scholar, and CINAHL, using the following search terms: (COVID-19 OR SARS-CoV-2* OR Severe Acute Respiratory Syndrome Coronavirus 2* OR COVID*) AND (Psychosis) AND (Adolescent OR Children OR Teenager). An updated search was completed on 10 August 2024. Search results from six databases were manually checked to remove any duplication. The extracted data was then arranged in a standardised template. The extracted data included: demographic characteristics of the patients including age, gender, ethnicity, past personal and family psychiatric history, clinical features including neurological and psychotic symptoms and management including outcome.

**Results:** This descriptive systematic review identified 15 cases of incident psychosis in patients with antecedent or concurrent Covid-19. Out of 15 cases, 9 were males, 4 were females and 2 did not report any sex. The mean age of patients in our sample was 15.1 years with 2 cases not reporting the actual age. Delusions were present in all cases (100%) of patients, whereas hallucinations were reported in only 33% of the cases. Disorganised speech or behaviour was reported in 40% of the cases. Psychotic symptoms lasted from approximately 7–90 days. Family history was positive for 2/15 cases (7.5%) with psychosis and BPAD respectively. Only 2 cases had past personal history of mental illness (Depression and anxiety). All patients received anti-psychotic medications as a part of the treatment, whereas 33% patients received intravenous immunoglobulins concurrently with antipsychotic medication. Full remission was obtained in nearly all cases after treatment.

**Conclusion:** Covid-19 related psychosis differs in various aspects and should be considered as a separate entity when considering the assessment and management. It differs in many ways from a typical early onset psychotic episode both in presentation and treatment response.

